# Matrine inhibits the development and progression of ovarian cancer by repressing cancer associated phosphorylation signaling pathways

**DOI:** 10.1038/s41419-019-2013-3

**Published:** 2019-10-10

**Authors:** Xi Zhang, Guoqing Hou, Andong Liu, Hui Xu, Yang Guan, Yaosong Wu, Jie Deng, Xuan Cao

**Affiliations:** 1 Department of Medical Genetics, School of Basic Medicine, Tongji Medical College, Huazhong University of Science and Technology, Wuhan, 430030 China; 2 Ultrastructural Pathology Laboratory, Department of Pathology, School of Basic Medicine, Tongji Medical College, Huazhong University of Science and Technology, Wuhan, 430030 China; 3 Ultrastructural Pathology Center, Renmin Hospital of Wuhan University, Wuhan, 430060 China; 4 The Institute of Cancer Molecular Mechanisms & Drug Targets, Henan University of Traditional Chinese Medicine, Zhengzhou, 450046 China

**Keywords:** Cancer therapy, Small molecules

## Abstract

Ovarian cancer remains the most lethal gynecologic malignancy with late detection and acquired chemoresistance. Advanced understanding of the pathophysiology and novel treatment strategies are urgently required. A growing body of proteomic investigations suggest that phosphorylation has a pivotal role in the regulation of ovarian cancer associated signaling pathways. Matrine has been extensively studied for its potent anti-tumor activities. However, its effect on ovarian cancer cells and underlying molecular mechanisms remain unclear. Herein we showed that matrine treatment inhibited the development and progression of ovarian cancer cells by regulating proliferation, apoptosis, autophagy, invasion and angiogenesis. Matrine treatment retarded the cancer associated signaling transduction by decreasing the phosphorylation levels of ERK1/2, MEK1/2, PI3K, Akt, mTOR, FAK, RhoA, VEGFR2, and Tie2 in vitro and in vivo. Moreover, matrine showed excellent antitumor effect on chemoresistant ovarian cancer cells. No obvious toxic side effects were observed in matrine-administrated mice. As the natural agent, matrine has the potential to be the targeting drug against ovarian cancer cells with the advantages of overcoming the chemotherapy resistance and decreasing the toxic side effects.

## Introduction

Ovarian cancer is the most lethal gynecologic malignancy with late-stage presentation at the time of diagnosis^1,2^. Epithelial ovarian cancer (EOC) makes up about 90% of all ovarian cancers and accounts for most deaths from this disease^3,4^. For EOC in the past decades, the standard treatment is cytoreductive surgery and platinum-based chemotherapy^5,6^. Although most patients respond well to this initial treatment, recurrences are frequently to occur within a median of 16 months for patients with advanced stage for the development of chemoresistance^4,7^. Therefore, advanced understanding of the pathophysiology and novel treatment strategies are required to conquer the ovarian cancer.

Rapid development and utilization of high-throughput sequencing technology has greatly advanced the understanding of the mechanisms of tumorigenesis at the DNA level, such as variations among DNA, copy number, and mRNA/protein expression^8^. However, more and more proteomic investigations reveal that complex rewriting of signaling pathways, mediated by the protein post-translational modifications (PTMs) such as phosphorylation, plays a critical role in the development of ovarian cancer and its chemoresistance^8–11^.

Matrine is a natural quinolizidine alkaloid component extracted from the Sophora root, which is a traditional herb widely used in China, Japan and European countries^12,13^. Matrine was reported to exhibit various pharmacological activities to treat inflammation, liver fibrosis, viral hepatitis, arrhythmia, and other diseases for centuries^14–19^. Recently, matrine has been extensively studied for its potent anti-cancer activities via binding to its possible targets and blocking the cancer associated signaling pathways in various cancer cells^20–27^. As a natural anti-cancer agent, matrine inhibits cell proliferation and arrests cell cycle progression through up-regulating cyclin-dependent kinase (CDK) inhibitors p21 and p27 and down-regulating cyclin D1 protein in human retinoblastoma and melanoma cells^25,28^. It has been reported that matrine induces apoptosis by up-regulating Bax and down-regulating Bcl-2, inhibiting PI3K/AKT pathway in ^V600E^BRAF harboring melanoma cells, or by decreasing the phosphorylation levels of Akt and ERK1/2 in human acute myeloid leukemia cells^25,26,29,30^. Matrine also suppresses cancer cell invasion via down-regulation of EGFR/Akt/MMP-9, Ras/ERK, TGF-/Smad, Wnt signaling pathways^31–33^. Moreover, matrine restrains angiogenesis by reducing the expression of VEGF, EGF, VEGFR1, MMP-9, and MMP-2 in human non-small cell lung cancer (NSCLC) A549 cells and MDA-MB-231 cells^21,30^. However, the effects of matrine on ovarian cancer cells and its underlying molecular mechanisms have not been investigated.

In this study, we used epithelial ovarian cancer cells A2780, SKOV3 and cisplatin-resistant cells A2780/DDP to examine the therapeutic potential of matrine and explored its anti-cancer mechanisms by regulating proliferation, apoptosis, autophagy, invasion and angiogenesis via repressing cancer associated phosphorylation signaling pathways.

## Results

### Matrine inhibits the proliferation of ovarian cancer cells via downregulating MAPK/ERK pathway

**Fig. 1:**
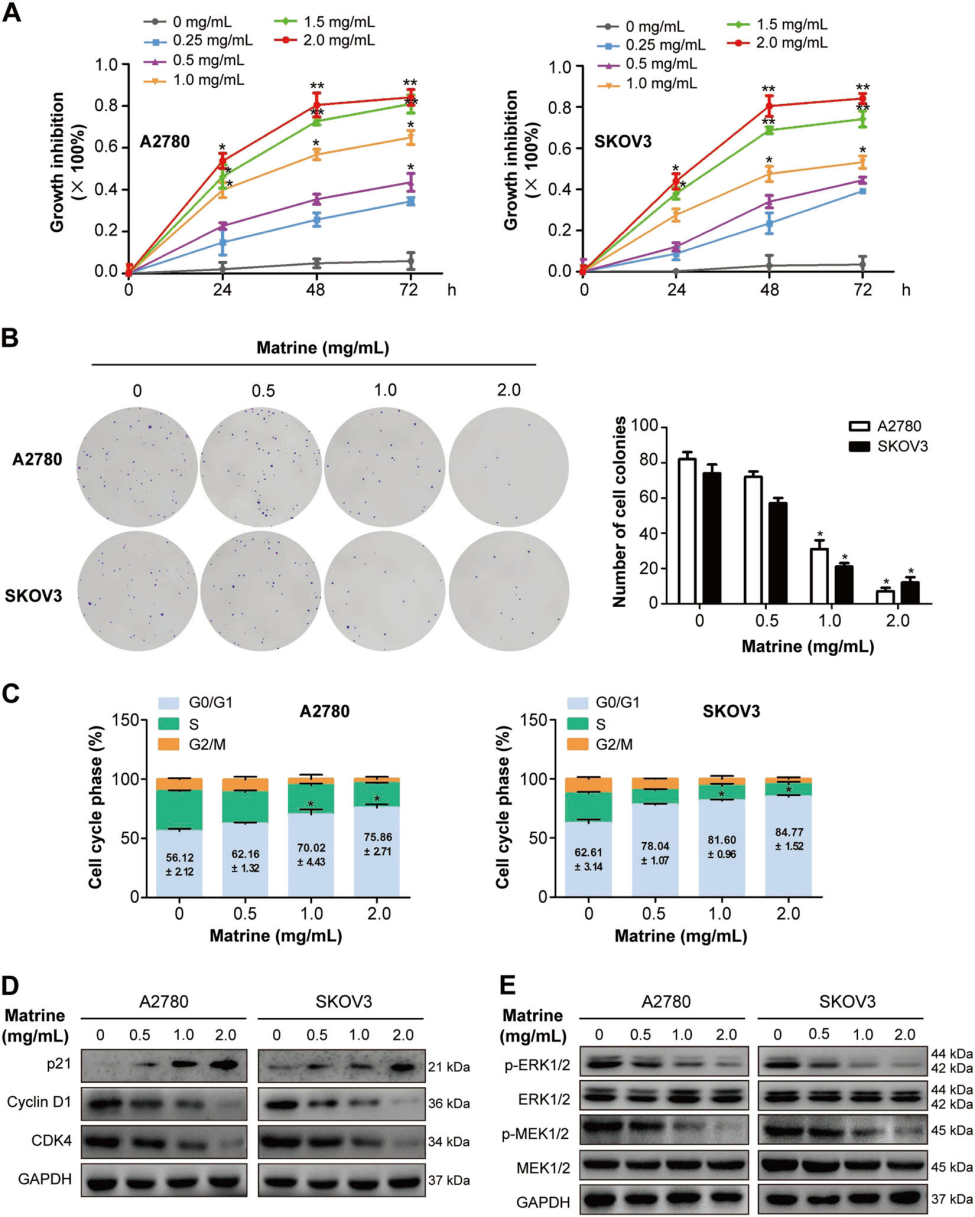
Matrine inhibits the proliferation of ovarian cancer cells. **a** A2780 and SKOV3 cells were treated with matrine ranging from 0–2.0 mg/mL for 24, 48, and 72 h, followed by MTT assay to evaluate cell growth. Data are presented as mean ± SD (*P *<* 0.05, **P *<* 0.01). **b** Representative images of colony formation assay showing colonies formed by A2780 and SKOV3 cells incubated with matrine from 0 to 2.0 mg/mL. Graph indicating the number of colonies formed in A2780 and SKOV3 cells with matrine treatment. Results are representative of three experiments (*P *<* 0.05). **c** Cell cycle analysis was measured by the percentage of cells in each phase in matrine-treated A2780 and SKOV3 cells. **d** A2780 and SKOV3 cells were incubated with matrine for 24 h. The expression levels of p21, cyclin D1 and CDK4 were measured by western blot. **e** Matrine reduced the phosphorylation levels of ERK1/2 and MEK1/2 in A2780 and SKOV3 cells. GAPDH was used as a loading control

To detect the inhibitory effect of matrine on the proliferation of ovarian cancer cells, A2780 and SKOV3 cells were examined by MTT assay. As shown in Fig. 1a, matrine treatment significantly inhibited the proliferation of A2780 and SKOV3 cells in a dose- and time-dependent manner. To evaluate the reduction in cellular viability, colony formation assay was performed^34^. Compared with the control group, the clonogenic ability of A2780 and SKOV3 cells was drastically decreased after incubated with 2.0 mg/mL matrine (Fig. 1b). These data proved that matrine treatment restrained proliferation of ovarian cancer cells in vitro.

To further investigate the mechanisms by which matrine suppressed the growth of ovarian cancer cells, A2780 and SKOV3 cells were treated with various concentrations of matrine for 24 h, and then cell cycle analysis was conducted. Matrine treatment prominently induced a dose-dependent increase in the percentage of cells in G0/G1 phase (from 56.12 ± 2.12% to 75.86 ± 2.71% in A2780; from 62.61 ± 3.14% to 84.77 ± 1.52% in SKOV3), accompanied by a corresponding decrease in the S and G2/M phase in A2780 and SKOV3 cells (Fig. 1c). We then examined the cell cycle related check point proteins that controlled G1/S phase transition^35^. P21, a cyclin dependent kinase inhibitor, could lead to G0/G1 arrest by inhibiting the cyclin D1 and CDK4/6 activities^36^. Western blot analysis showed that matrine treatment dramatically increased the expression of p21, but largely reduced the protein expression of cyclin D1 and CDK4 in A2780 and SKOV3 cells (Fig. 1d). MAPK/ERK pathway plays a critical role in the proliferation of cancer cells^37^. Matrine treatment drastically reduced the phosphorylation levels of ERK1/2 and MEK1/2 in A2780 and SKOV3 cells (Fig. 1e), which are the downstream kinases of the EGFR signaling pathway^34^.

### Matrine induces the apoptosis of ovarian cancer cells through suppressing PI3K/Akt signaling pathway

**Fig. 2:**
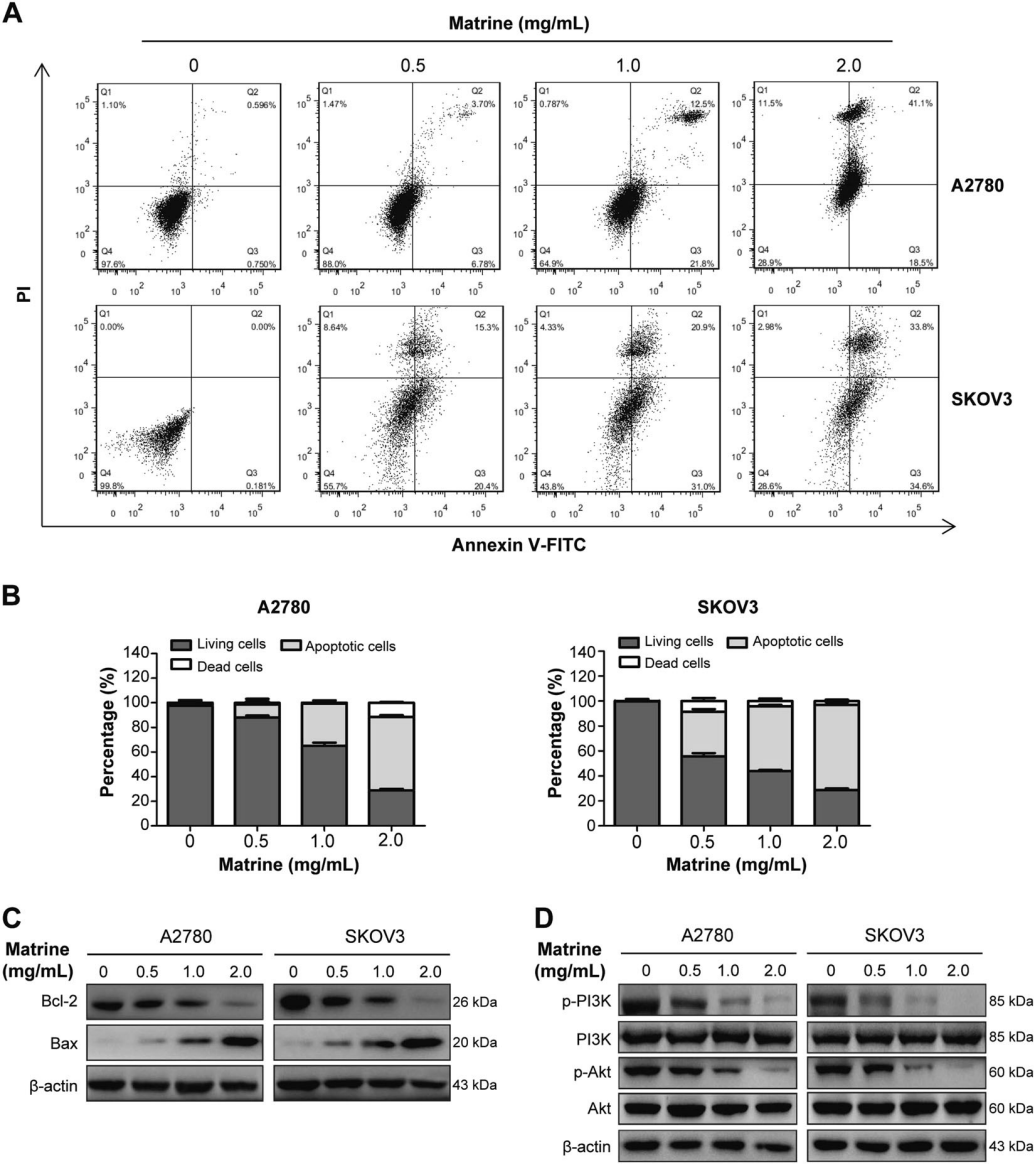
Apoptosis induced by matrine in ovarian cancer cells. **a** Cells were measured by flow cytometry analysis of annexin V/PI double staining in A2780 and SKOV3 cells treated with various concentrations of matrine for 24 h. Region Q1 shows dead cells, Q2 shows late apoptotic cells, Q3 shows early apoptotic cells and Q4 shows living cells. Q2 and Q3 are collectively called apoptotic cells. **b** The distribution and percentage of living, apoptotic and dead cells for A2780 and SKOV3 cells were detected by flow cytometry. **c** A2780 and SKOV3 cells were treated with different concentrations of matrine for 24 h. Bcl-2 and Bax expression at protein level were evaluated by western blot. **d** The phosphorylation levels of PI3K and Akt in A2780 and SKOV3 cells were decreased after treatment of matrine. β-actin was used as an internal control

During our preliminary trials, the phenomenon of cell death after treated with matrine was often observed, and the amount of dead cells increased at the dose- and time-dependent manner. To investigate whether matrine induces apoptosis of ovarian cancer cells, we detected the apoptosis rate by flow cytometry analysis with annexin V/PI double staining in A2780 and SKOV3 cells exposed to various concentrations of matrine for 24 h. The results showed that the ratio of apoptotic cells (Q2 + Q3, Q2 and Q3 represent late and early apoptotic cells, respectively) significantly increased as the concentration of matrine increasing from 0 mg/mL to 2.0 mg/mL. As revealed by flow cytometry analysis, the percentage of apoptotic cells reached 59.6% (Q2:41.1%, Q3:18.5%) and 68.4% (Q2:33.8%, Q3:34.6%) for A2780 and SKOV3 cells treated with 2.0 mg/mL matrine (Fig. 2a, b). These results suggested that matrine facilitated cell apoptosis, which caused cell death after matrine incubation in vitro. It is well known that Bcl-2 and Bax play a pivotal role in the regulation process of apoptosis^34,38^. The results of western blot analysis showed that the expression of Bcl-2 was decreased and Bax was increased after treating with 2.0 mg/mL matrine for 24 h, indicating that matrine could promote apoptosis in A2780 and SKOV3 cells (Fig. 2c). As the upstream regulators of Bax and Bcl-2, PI3K/Akt signaling pathway was reported to play an important role in apoptosis. As expected, matrine treatment at the concentration of 2.0 mg/mL for 24 h significantly reduced the phosphorylation levels of PI3K and Akt without effect on their expression levels (Fig. 2d).

### Matrine triggers the autophagy of ovarian cancer cells by attenuating Akt/mTOR signaling pathway

**Fig. 3:**
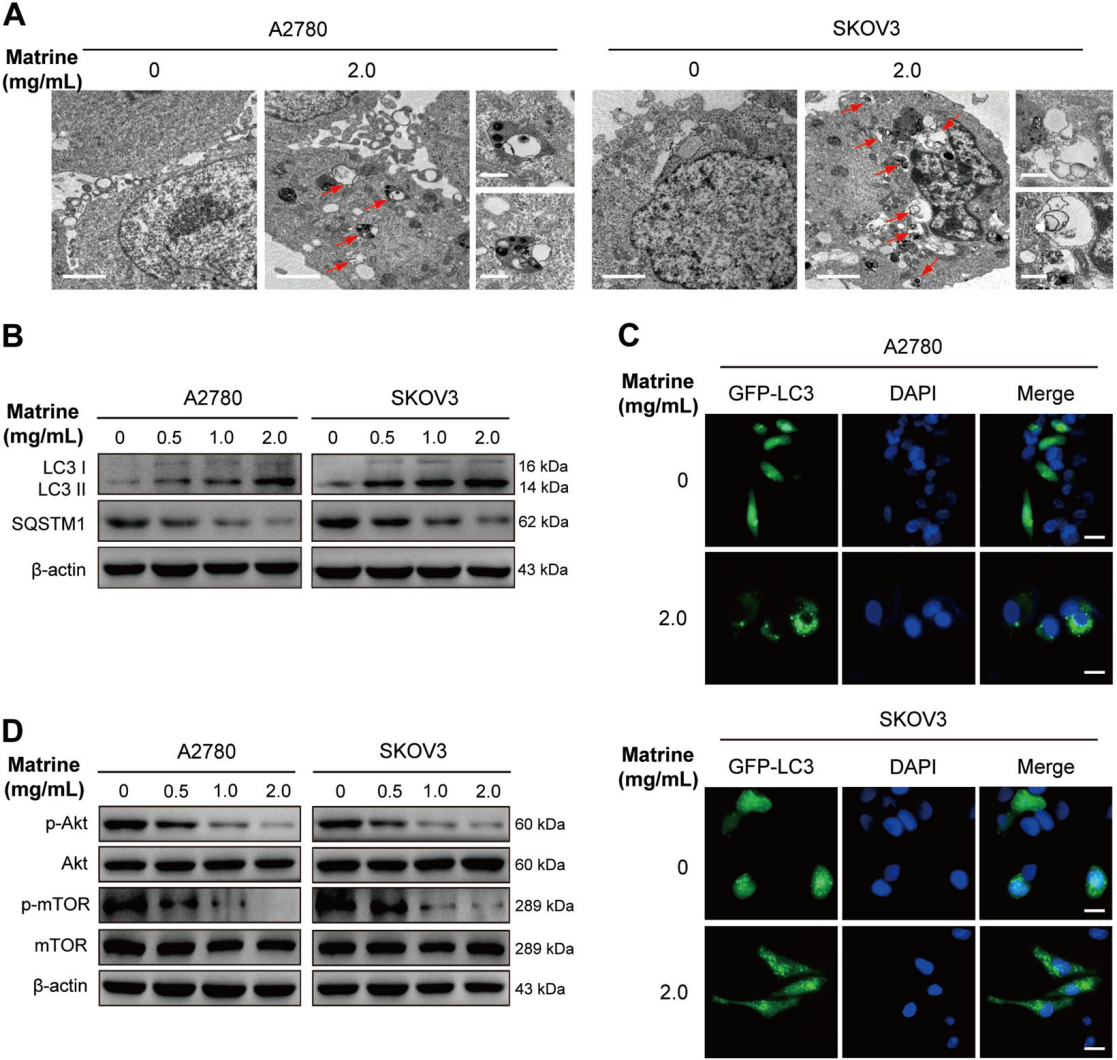
Matrine induces the autophagy of ovarian cancer cells. **a** After exposure to matrine at 2.0 mg/mL, representative transmission electron microscopic images were obtained from matrine-treated cells as compared with control. The autophagosomes or autolysosome-like structures were indicated at the red arrows (scale bar: 2 μm). On the right panel were images with higher magnifications (scale bar: 0.5 μm). **b** Expressions of LC3-I/II and SQSTM1/p62 in matrine-treated A2780 and SKOV3 cells were determined by western blot. **c** A2780 and SKOV3 cells were transfected with GFP-LC3 plasmid for 24 h followed by exposure to matrine for 24 h. The representative images of GFP-LC3 puncta were viewed to monitor autophagosome formation by fluorescence microscopy. Each image is the representation of three independent experiments. Scale bar: 20 μm. **d** Western blot of the phosphorylation levels of Akt and mTOR in matrine-treated cells compared to the untreated cells. β-actin was used as a control

Autophagy has an essential role in various pathophysiological processes, including tumorigenensis^39^. The role of autophagy in cancer is complex. Autophagy can contribute to cancer by promoting tumor cell survival under nutrient stress^40^. However, recent researches have shown that autophagy is more likely to be a tumor suppressor. The strategy to induce autophagy and enhance its tumor suppression attributes may serve as an anticancer therapy^41^. Matrine treatment has been reported to induce or inhibit autophagy in several kinds of cancer cells^23,42,43^, however it is unclear in ovarian cancer cells. To determine whether matrine regulates autophagy in ovarian cancer cells, we observed the autophagosomes and autolysosomes by using the transmission electron microscopy technique. As shown in Fig. 3a, the number of autophagosomes and autolysosomes markedly increased in A2780 or SKOV3 cells treated with 2.0 mg/mL matrine, but not in the control group. Furthermore, we examined the levels of LC3-I/II, a well-known autophagosomal marker^44^. LC3-II levels significantly increased in matrine-treated A2780 or SKOV3 cells compared to β-actin loading control. At the same time, we assessed the levels of SQSTM1/p62, a widely accepted autophagic substrate^45^. Expression levels of SQSTM1/p62 were markedly decreased by matrine treatment in A2780 or SKOV3 cells (Fig. 3b). These results strongly indicated that matrine treatment induced autophagy in ovarian cancer cells. This effect was also confirmed by the observation that 2.0 mg/mL matrine treatment markedly increased the number of GFP-LC3-positive puncta in A2780 or SKOV3 cells transiently transfected with GFP-LC3 constructs (Fig. 3c). Akt/mTOR signaling pathway is related to various physiological processes, including autophagy^44^. To investigate whether the Akt/mTOR signaling pathway is involved in matrine-induced autophagy, we evaluated the expression and the phosphorylation levels of Akt and mTOR. Matrine treatment showed no effect on the expression levels of Akt and mTOR, but the phosphorylation levels of them were significantly inhibited by matrine in a dose-dependent manner in A2780 or SKOV3 cells (Fig. 3d). These findings supported that matrine promoted autophagy via negatively regulating the phosphorylation of Akt/mTOR signaling pathway.

### Matrine suppresses the migration and invasion of ovarian cancer cells by inactivating FAK and RhoA

**Fig. 4:**
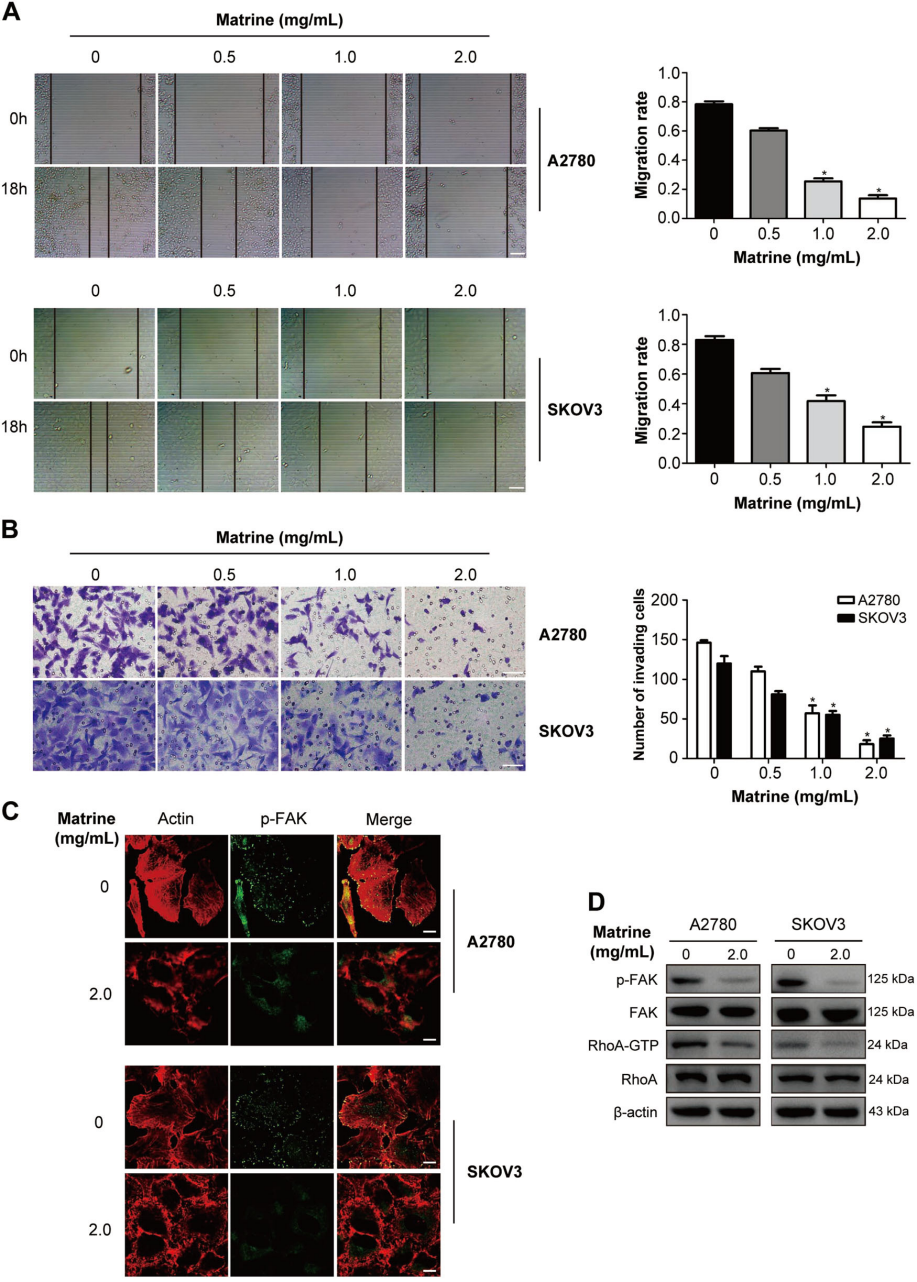
Matrine suppresses the migration and invasion of ovarian cancer cells. **a** The scratch wound-healing assay was performed to evaluate the effect of matrine on cell migration. A2780 and SKOV3 cells were treated with the indicated concentration of matrine for 18 h. Representative images of cell migration of A2780 and SKOV3 cells in a scratch wound-healing assay were shown in the left panel. Scale bar: 50 μm. The bar graphs showed mean ± SD of migration rate of wounds for A2780 and SKOV3 cells from three independent experiments (right panel) (*P *<* 0.05). **b** Transwell assay was performed to evaluate the effect of matrine on cell invasion. A2780 and SKOV3 cells were treated with the indicated concentrations of matrine for 18 h. Shown are representative images of cell invasion of A2780 and SKOV3 cells (left panel). The bar graphs showed mean ± SD of the numbers of invading cells for A2780 and SKOV3 cells from three independent experiments (right panel) (*P *<* 0.05). Scale bar: 20 μm. **c** Matrine treatment in A2780 and SKOV3 cells led to the decrease of the formation of actin stress fibers and focal adhesions at the cell bottom. Images shown are representative of three independent experiments. Scale bar: 10 μm. **d** The levels of active FAK (p-FAK) and RhoA (RhoA-GTP) in A2780 and SKOV3 cells were down-regulated after treatment of matrine. β-actin was used as an internal control

Cancer metastasis is the main cause of mortality in patients with cancer. To examine whether matrine may repress the migration of ovarian cancer cells, the wound healing assay was performed. Results showed that matrine apparently suppressed the migration of A2780 and SKOV3 cells in a dose-dependent manner (Fig. 4a). Furthermore, transwell assay with matrigel was carried out to evaluate the effect of matrine on the invasion ability of A2780 and SKOV3 cells. Number of invading cells through the permeable membrane of the matrine-treated group was drastically less than that of the control group (Fig. 4b). These data indicated that the migration and invasion of A2780 and SKOV3 cells was inhibited by matrine. The Rho family of small GTPases plays an essential role in cell migration and invasion by promoting cytoskeletal reorganization^46^. Active RhoA and focal adhesion kinase (FAK) are required for the formation of actin stress fibres and focal adhesions which are necessary for cell motility^47^. The confocal imaging results showed that exposure of A2780 and SKOV3 cells to 2.0 mg/mL matrine dramatically attenuated the formation of actin stress fibres and focal adhesions (Fig. 4c). Further western blot analysis revealed that matrine treatment remarkably decreased the levels of active FAK (p-FAK) and RhoA (RhoA-GTP) in A2780 and SKOV3 cells (Fig. 4d).

### Matrine impairs the tumor angiogenesis in vitro by blocking VEGF/VEGFR2 and ANG-1/Tie2 signaling pathways

**Fig. 5:**
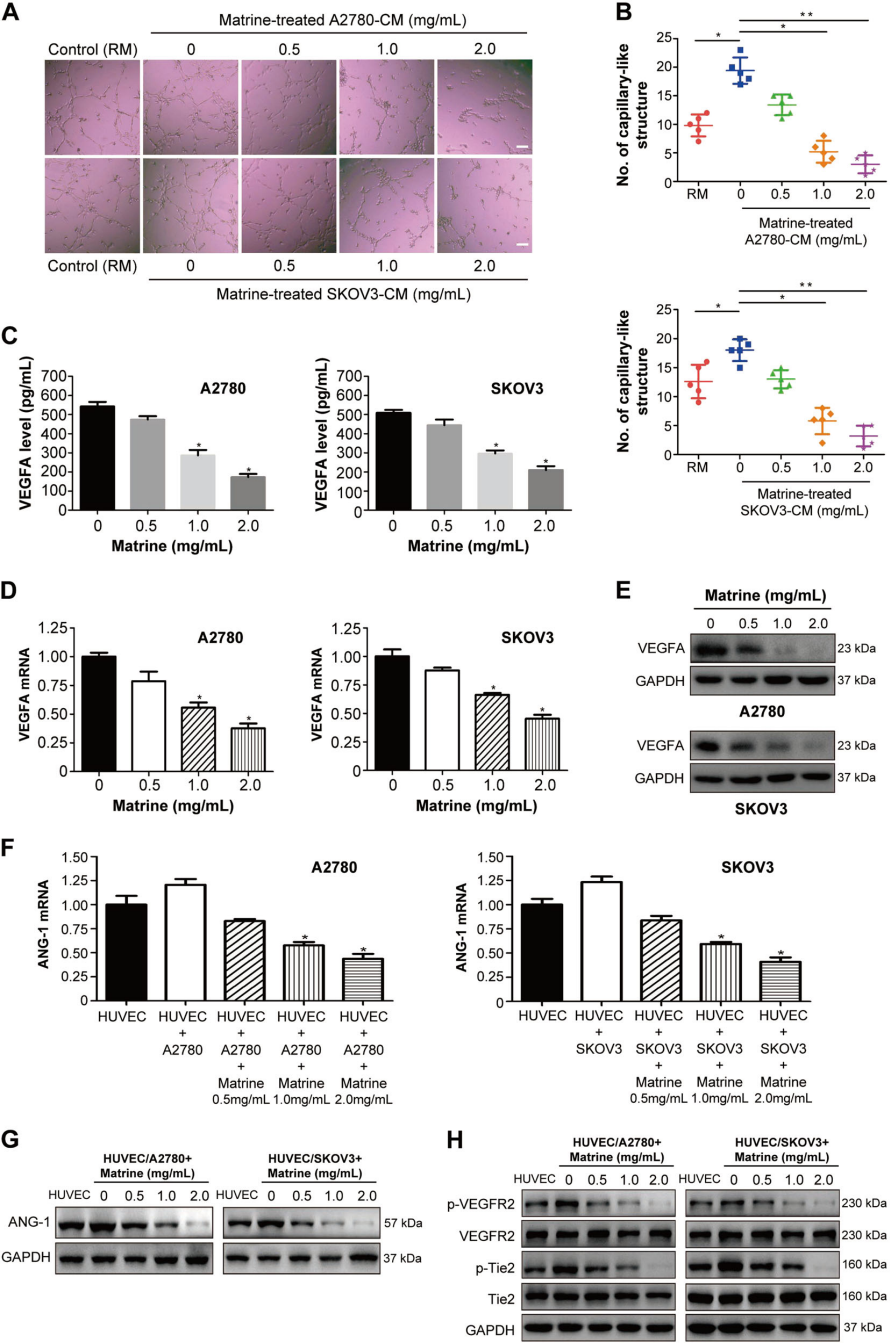
Effect of matrine on tumor angiogenesis in vitro. **a** Representative images of capillary-like structures. HUVECs were seeded on Matrigel in presence of regular media (RM) or A2780 and SKOV3 cell derived CM or matrine-treated A2780 and SKOV3 cell derived CM. RM served as control set. Scale bar: 20 μm. **b** The error-bar graphs showed the mean numbers of capillary-like structures formed by HUVECs in different experimental conditions (*P < 0.05, **P < 0.01). **c** The levels of VEGFA in the media of A2780 and SKOV3 cells treated with matrine were measured by ELISA. Data are represented as means ± SD of three independent experiments (*P *<* 0.05). **d** Effect of matrine on the mRNA levels of VEGFA in A2780 and SKOV3 cells. **e** The protein expressions of VEGFA were significantly decreased in A2780 and SKOV3 cells with the addition of matrine. **f** Effect of matrine on the mRNA levels of ANG-1 in HUVECs co-cultured with A2780 or SKOV3 cells. **g** The protein expressions of ANG-1 were decreased by matrine in HUVECs co-cultured with A2780 or SKOV3 cells. **h** The phosphorylation levels of VEGFR2 and Tie2 were down-regulated by matrine in HUVECs co-cultured with A2780 or SKOV3 cells. GAPDH was used as a loading control

Tumor angiogenesis is a vital process to supply adequate oxygen and nutrition to cancer cells in the survival and growth of tumor^48^. To examine the effect of matrine on tumor angiogenesis, in vitro angiogenesis assays were carried out. The results suggested that ovarian cancer cell derived conditioned media (CM) significantly induced HUVEC to form capillary-like structures compared to the control group with regular media (RM), whereas matrine treatment notably impaired the stimulation effect induced by the ovarian cancer cell-CM (Fig. 5a, b). Vascular endothelial growth factors (VEGFs) and angiopoietins play a crucial role in promoting tumor angiogenesis^49,50^. Aberrant expression and release of VEGFs has been reported in many types of cancer cells. The experiments were conducted to examine whether matrine treatment could regulate the VEGFs expression of the ovarian cancer cells. The concentrations of VEGFA, the typical member of VEGF family, in the tumor cell culture media were measured by enzyme linked immunosorbent assay (ELISA). The levels of VEGFA secreted into the media were significantly reduced after matrine treatment in a dose-dependent manner in A2780 and SKOV3 cells (Fig. 5c). Furthermore, the results showed that the mRNA levels and protein expressions of VEGFA were significantly decreased in A2780 and SKOV3 cells with the addition of 2.0 mg/mL matrine (Fig. 5d, e). VEGF stimulates angiogenesis via binding VEGF receptor (VEFGR) and inducing its tyrosine phosphorylation in endothelial cells^51^. Angiopoietins, the endothelial-produced proteins, modulate vessel stability through binding the tyrosine kinase Tie2^52^. Although four members (ANG-1 to ANG-4) have been found in angiopoietin family, ANG-1 is a Tie2 receptor agonist by inducing its tyrosine phosphorylation^52^. Therefore, we sought to determine whether matrine could affect the expression level of ANG-1 and the tyrosine phosphorylation status of VEGFR2 and Tie2 in endothelial cells. HUVECs were seeded on the bottom wells and co-cultured together with the ovarian cancer cells on the permeable membrane by utilizing Falcon 6-multiwell plates and Falcon cell culture inserts^51^. The mRNA expressions of ANG-1 in HUVECs were upregulated during the HUVEC/A2780 or HUVEC/SKOV3 co-culture relative to the HUVECs monoculture (Fig. 5f). With the addition of 2.0 mg/mL matrine into A2780 or SKOV3 cells in the upper compartment, the mRNA levels and protein expressions of ANG-1 in HUVECs were significantly downregulated, whereas VEGFR2 and Tie2 expression levels were not obviously influenced (Fig. 5f–h). Interestingly, the tyrosine phosphorylation levels of VEGFR2 and Tie2 in HUVECs were found to dramatically decline after the treatment of 2.0 mg/mL matrine (Fig. 5h). All these data demonstrated that matrine treatment notably repressed the tumor angiogenesis by attenuating the VEGF/VEGFR2 and ANG-1/Tie2 signaling pathways.

### Matrine inhibits the growth and metastasis of A2780 cells in vivo

**Fig. 6:**
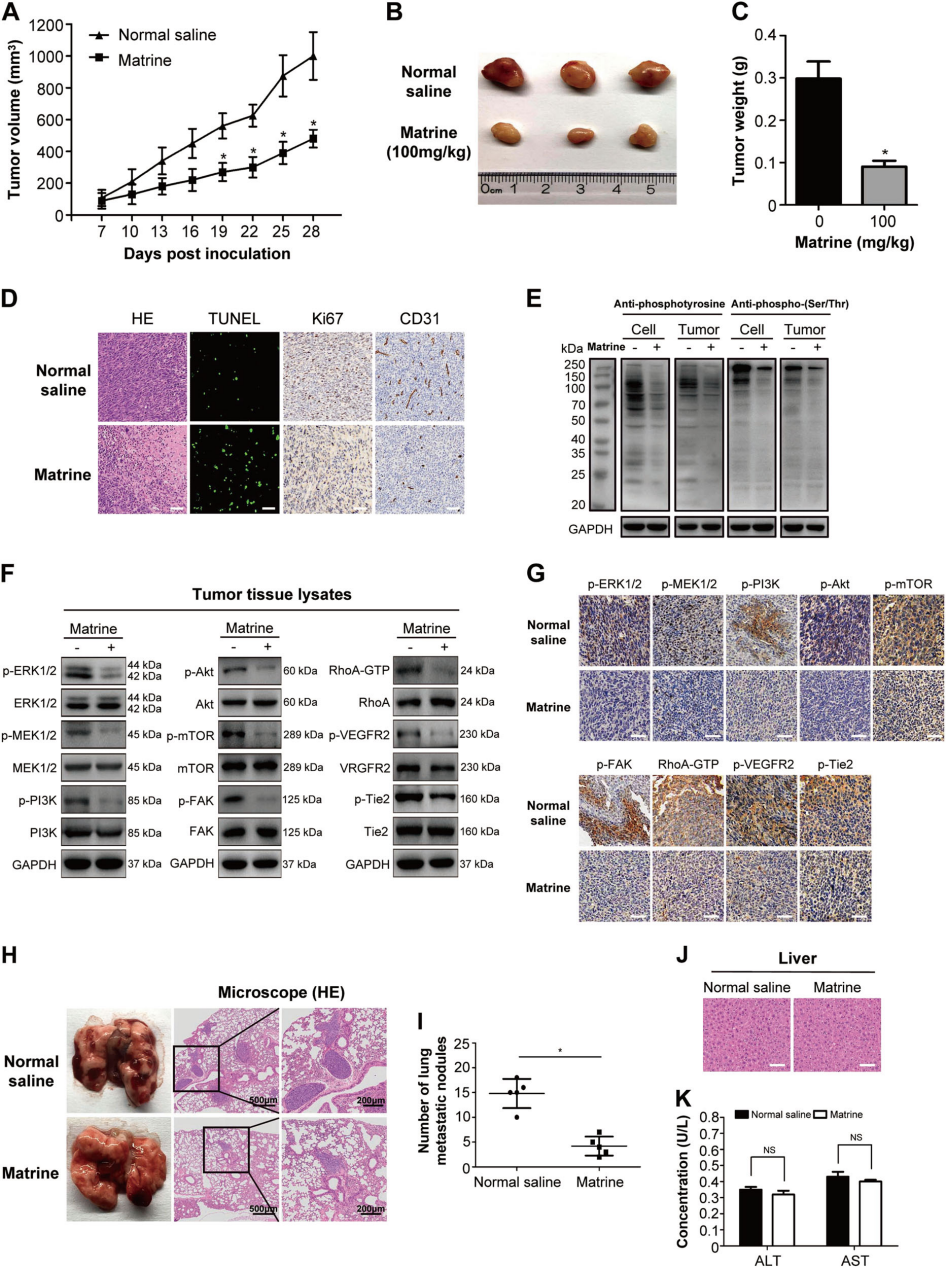
Inhibition of xenograft tumor growth and metastasis by matrine in vivo. **a** Time course of tumor growth, measured as tumor volume in each group at the indicated time of treatment with normal saline or matrine (100 mg/kg, ip). Data are presented as mean ± SD (*n* = 3, *P *<* 0.05). **b** Tumors in matrine-treated mice were significantly smaller than those in normal saline-treated mice. **c** The bar graph represented the mean of tumor weight from matrine-treated and control mice (*n* = 3, *P *<* 0.05). **d** The representative histological examinations of the dissected tumors with matrine or normal saline treatment using H&E staining, TUNEL assay, Ki-67 and CD31 antibodies. Scale bar: 50 μm. **e** The global phosphorylation levels of tyrosine and serine/threonine were significantly decreased in matrine-treated A2780 cells and in matrine-treated tumor tissue lysates, respectively. **f**, **g** Western blot and IHC staining demonstrated a decrease in the phosphorylation levels of ERK1/2, MEK1/2, PI3K, Akt, mTOR, FAK, RhoA, VEGFR2, and Tie2 in xenografts treated with matrine compared with those in control group. Scale bar: 50 μm. **h** A2780 cells were injected into tail veins of BALB/c nude mice. Representative images showed lungs with metastatic nodules from the matrine-treated mice and the control group. The left panel presents macroscopic appearances of lung metastatic nodules, and the middle and right panels present the H&E staining of lung tissues in mice with matrine or normal saline treatment. **i** The number of lung metastatic nodules was significantly reduced in mice treated with matrine compared with the control group. **j** H&E staining of hepatic tissues in mice with matrine or normal saline treatment. Scale bar: 50 μm. **k** Concentrations of ALT and AST were measured in the blood at the end of matrine treatment. Data are presented as mean ± SD

To investigate the role of matrine in vivo, xenograft model was established by subcutaneous injection of A2780 cells in the flank of BALB/c nude mice^34,35^. Since the 7th day, the mice were treated with an intraperitoneal injection of 100 mg/kg matrine or normal saline thrice a week for 3 weeks. Tumor volume, size and weight of mice in matrine group were found to be much smaller than those of normal saline group. The results demonstrated that matrine resulted in pronounced suppression of A2780 tumor growth (Fig. 6a–c). Haematoxylin and eosin (H&E) staining was utilized to observe the changes of tumor cell morphology^34^. The images illustrated matrine caused high level of necrosis lesions in tumors. The sections of tumors were assayed for apoptosis analysis using a TUNEL kit that labels apoptotic nuclei with a green fluorescent marker. The incidence of apoptosis in matrine-treated group was conspicuously higher than normal saline group. Immunohistochemistry (IHC) analysis showed that treatment with matrine resulted in a remarkably smaller proportion of Ki-67 (proliferation marker protein) and CD31 (antigen involved in angiogenesis) positive ovarian carcinoma cells in tumors compared with the control group (Fig. 6d). Furthermore, we checked the total phosphorylation levels of the tumor tissue lysates^53^. As shown in Fig. 6e, the global phosphorylation levels of tyrosine and serine/threonine were significantly attenuated in the matrine-treated tumor tissue lysates, which is consistent with the situation of ovarian cancer A2780 cells exposed to matrine in vitro. Subsequently, we examined the expression and the phosphorylation levels of ERK1/2, MEK1/2, PI3K, Akt, mTOR, FAK, RhoA, VEGFR2 and Tie2 in tumor tissue lysates. Similar to the data from A2780 cells in vitro, matrine administration obviously decreased the phosphorylation levels of ERK1/2, MEK1/2, PI3K, Akt, mTOR, FAK, RhoA, VEGFR2 and Tie2 in vivo, but showed no effect on their expression levels (Fig. 6f). Meanwhile, IHC staining confirmed that the phosphorylation levels of these proteins were significantly decreased in tumors with matrine treatment compared with the control group (Fig. 6g).

To examine the effect of matrine on ovarian cancer cell metastasis, A2780 cells were injected into tail veins of BALB/c nude mice. Six weeks after mice were inoculated, metastases were distributed in the lung parenchyma as nodules. The average number of lung metastatic nodules in mice treated with matrine was significantly decreased compared with the control group (Fig. 6h, i). These results suggested that matrine can efficiently inhibit the metastasis of A2780 ovarian cancer cells. Intriguingly, hepatic tissues of matrine-treated mice showed normal cellular morphology as the control group (Fig. 6j). Moreover, there was no obvious difference in serum activity of alanine transaminase (ALT) and aspartate transaminase (AST) between marine-treated and control mice (Fig. 6k). Collectively, all these data strongly indicated that matrine could be an effective and safe therapy to ovarian cancer in vivo.

### Matrine exhibits antitumor effect on ovarian cancer cells with chemoresistance

**Fig. 7:**
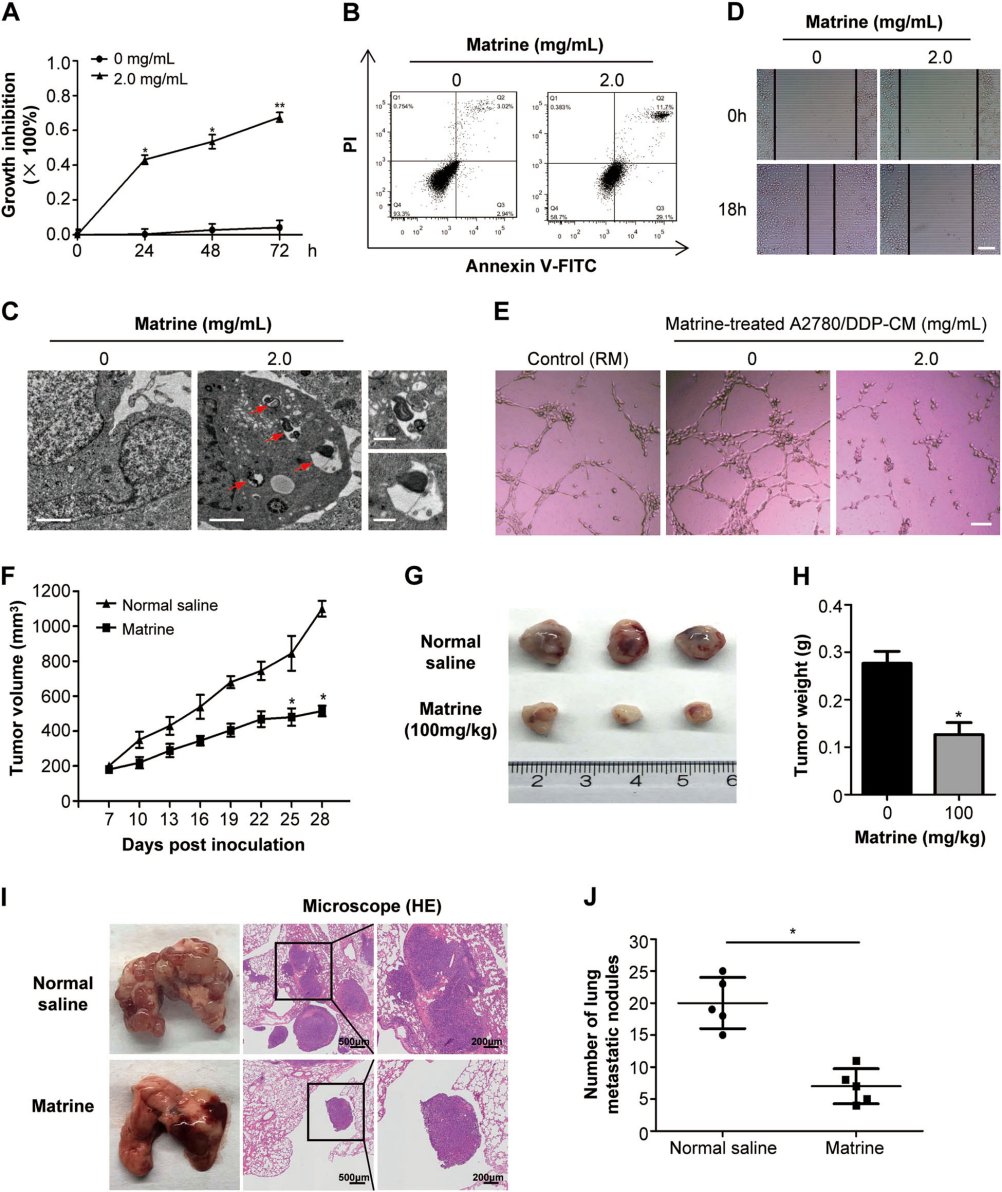
The antitumor effects of matrine on A2780/DDP in vitro and in vivo. **a** The proliferation inhibition of matrine in A2780/DDP cells was determined by MTT assay. Data are presented as mean ± SD from three independent experiments (*P *<* 0.05, **P *<* 0.01). **b** Changes in apoptosis in matrine treated A2780/DDP cells were determined by flow cytometry analysis. **c** Autophagosome and autolysosome vesicles of A2780/DDP cells treated with 2.0 mg/mL matrine for 24 h were visualized by transmission electron microscopy. The typical images of autophagosomes and autolysosomes were indicated at the red arrows (scale bar: 2 μm). On the right panel were images with higher magnifications (scale bar: 0.5 μm). **d** Wound healing assay was performed to examine the effect of matrine on the migration of A2780/DDP cells. Scale bar: 50 μm. **e** Matrine inhibited the tube formation of HUVECs in Matrigel in presence of regular media (RM) or A2780/DDP cell derived CM or matrine-treated A2780/DDP cell derived CM. Scale bar: 20 μm. **f** Effect of matrine on the growth of A2780/DDP xenograft tumors. The changes of tumor volumes were shown between matrine and normal saline group (*n* = 3, *P *<* 0.05). **g** Tumors in matrine-treated mice were significantly smaller than those in normal saline group. **h** The bar graph represented the mean of tumor weight from matrine-treated and control mice (*n* = 3, *P *<* 0.05). **i** Representative images of lungs with metastatic nodules (left panel) and the corresponding images of H&E-stained lungs with metastases (middle and right panels). **j** The lung metastatic nodules were examined and quantified in mice treated with matrine and normal saline

Considering that matrine inhibits the development and progression of ovarian cancer through repressing the cancer associated phosphorylation signaling pathways, we speculated matrine treatment should have effect on ovarian cancer cells with chemoresistance. To verify this guess, A2780/DDP, cisplatin-resistant human ovarian cancer cell line, was tested in the experiments. The results showed that 2.0 mg/mL matrine treatment significantly inhibited the proliferation of A2780/DDP cells (Fig. 7a). At the same time, the induction of apoptosis (Fig. 7b) and autophagy (Fig. 7c) was observed in matrine-treated A2780/DDP cells. Furthermore, matrine treatment remarkably suppressed migration (Fig. 7d) and angiogenesis (Fig. 7e) of A2780/DDP cells. Consistent with above results in vitro, matrine administration obviously inhibited the growth of subcutaneous A2780/DDP xenograft tumors in nude BALB/c mice (Fig. 7f–h). Furthermore, the effect of matrine on the metastasis ability of A2780/DDP ovarian cancer cells was determined by injecting A2780/DDP cells into tail veins of nude mice. The results revealed a significant decrease in the number of metastatic nodules in lungs of mice treated with matrine compared with the control group (Fig. 7i, j). These results strongly proved that matrine showed antitumor effect on ovarian cancer cells with chemoresistance in vitro and in vivo.

## Discussion

Ovarian cancer remains the life-threatening disease of women even though the recent improvement from the therapies of platinum, bevacizumab and PARP inhibitor^2^. Novel molecular targeting agents are required to conquer this disease. Matrine has been reported to exhibit potent therapeutic effects through blocking cancer associated signaling pathways in various cancer cells^21–27^. The present study demonstrates that matrine inhibits the development and progression of ovarian cancer via regulating proliferation, apoptosis, autophagy, invasion and angiogenesis. Matrine treatment showed dose- and time-dependent growth inhibition by inducing an increase of G0/G1 phase and a decrease of S and G2/M phase in A2780 and SKOV3 cells and the molecular mechanisms were associated with the upregulation of p21 and the downregulation of cyclin D1 and CDK4. Matrine treatment was observed to exert the anti-cancer effect by inducing apoptotic and autophagic cell death in A2780 and SKOV3 cells. The expression of antiapoptotic protein Bcl-2 decreased and proapoptotic protein Bax increased in the matrine-induced apoptotic A2780 and SKOV3 cells. LC3-II, the autophagosomal marker, increased and SQSTM1, the autophagic substrate, decreased in matrine-induced autophagic A2780 and SKOV3 cells. Matrine suppressed the migration and invasion abilities of A2780 and SKOV3 cells by attenuating the formation of actin stress fibres and focal adhesions. Tumor angiogenesis was impaired by matrine treatment via regulating the expression of VEGFA of the ovarian cancer cells and ANG-1 of HUVECs co-cultured with A2780 and SKOV3 cells in vitro.

A growing body of proteomic investigations suggest that phosphorylation has a pivotal role in the regulation of ovarian cancer associated signaling pathways^4,8–11^. The levels of phosphorylated ERK1/2 and MEK1/2 were decreased by matrine treatment in A2780 and SKOV3 cells with the inhibition of proliferation. During the matrine-induced apoptosis and autophagy, the phosphorylation levels of PI3K, Akt and mTOR were examined to decrease in A2780 and SKOV3 cells. Matrine treatment decreased the levels of phosphorylated FAK and active RhoA (RhoA-GTP) of A2780 and SKOV3 cells with the suppression of migration and invasion. As for restraining angiogenesis in vitro, the phosphorylation levels of VEGFR2 and Tie2 in HUVECs co-cultured with A2780 and SKOV3 cells were observed to decline after matrine treatment.

Administration of matrine was found to inhibit the growth of subcutaneous A2780 xenograft tumors in nude BALB/c mice. The global phosphorylation levels of tyrosine and serine/threonine were reduced in the matrine-treated tumor tissue lysates. Matrine administration obviously decreased the phosphorylation levels of ERK1/2, MEK1/2, PI3K, Akt, mTOR, FAK, RhoA, VEGFR2 and Tie2 in vivo, and then accordingly retarded cancer associated signaling transduction. Metastasis of A2780 ovarian cancer cells was also efficiently inhibited by matrine treatment in nude mice. Owing to the mechanism of matrine as the anti-cancer drug is by means of repressing cancer associated phosphorylation signaling pathways^34^, which is distinct from that of the chemotherapy, it is rational to expect that matrine has an effect on chemoresistant ovarian cancer cells. As expected, matrine treatment showed excellent antitumor effect on A2780/DDP cells. The natural agents from the traditional herb are usually deemed to be safer than the chemical drugs^12^. Indeed, the matrine-treated mice showed no obvious toxic effects by the analysis of hepatic tissues and serum activity of ALT and AST.

Taken together, the present study demonstrated that matrine treatment suppressed the development and progression of ovarian cancer by regulating proliferation, apoptosis, autophagy, invasion and angiogenesis via targeting the cancer associated phosphorylation signaling pathways. As for the specific target or targets of matrine in ovarian cancer cells, further investigations need to be carried out to uncover it in future. As the natural agent, matrine has the potential for development into targeting drug against ovarian cancer with the advantages of overcoming the chemotherapy resistance and decreasing the toxic side effects.

## Materials and methods

### Cell lines and culture conditions

Human ovarian cancer cell lines SKOV3, A2780 and the cisplatin (DDP)-resistant subline A2780/DDP were obtained from Cobioer Biotechnology Co., Ltd. (Nanjing, China) and they were identified by STR profiling without mycoplasma contamination. Human umbilical vein endothelial cell (HUVEC) was obtained from American Type Culture Collection (ATCC, Manassas, VA). A2780, SKOV3 and A2780/DDP cell lines were maintained in RPMI1640 medium (Gibco, Carlsbad, CA, USA) supplemented with 10% fetal bovine serum (FBS, Invitrogen, Carlsbad, CA, USA) and 1% penicillin/streptomycin solution (Solarbio, Beijing, China). A2780/DDP cell line was cultured in RPMI1640 medium containing additional 1 μM cisplatin (Sigma-Aldrich, BP809) to maintain drug-resistance and further cultured in drug-free medium for one week before follow-up experiments. HUVECs were grown in endothelial cell medium (ECM, 5% FBS, 1% endothelial cell growth supplement and 1% penicillin/streptomycin solution) (ScienCell, San Diego, CA, USA). All the cells were incubated at 37°C in a humidified atmosphere containing 5% CO_2_.

### Drug preparation and reagents

Matrine was purchased from Xi’an Natural Field Bio-Technique Co., Ltd. (Xi’an, China), and dissolved in phosphate buffer saline (PBS) to a stock concentration of 100 mM. The solution was filtered through a 0.22 μm micropore filter and stored at −20 °C.

Antibodies against p21 (ab109520), cyclin D1 (ab134175), VEGFA (ab46154), ANG-1 (ab95230), phosphotyrosine (ab179530), phospho-(Ser/Thr) (ab17464), phospho-PI3K (Y607) (ab182651), phospho-mTOR (S2448) (ab109268) and phospho-FAK (S732) (ab4792) were purchased from Abcam (Cambridge, MA). Antibodies against CDK4 (A0016), Bcl-2 (A11025) and Bax (A12009) were obtained from ABclonal Biotechnology Co. (Wuhan, China). Antibodies against LC3I/II (#12741), SQSTM1/p62 (#5114), phospho-ERK1/2 (T202/Y204) (#4370), ERK1/2 (#4695), phospho-MEK1/2 (S221) (#2338), MEK1/2 (#9122), PI3K (#4292), phospho-Akt (S473) (#4060), Akt (#9272), mTOR (#2983), FAK (#3285), phospho-VEGF Receptor 2 (Y1175) (#2478), VEGF Receptor 2 (#2479), phospho-Tie2 (Y992) (#4221), Tie2 (#7403), β-actin (#4970) and GAPDH (#5174) were purchased from Cell Signaling Technology, Inc. (Beverly, MA). Antibodies against Ki67 (MA5-14520), CD31 (14-0319-82) were purchased from ThermoFisher Scientific (MA, USA). Anti-active RhoA-GTP (cat#26904) antibody was obtained from NewEast Bioscience (Wuhan, China). Anti-RhoA (sc-418) antibody was purchased from Santa Cruz Biotechnology (Santa Cruz, CA). HRP-conjugated anti-mouse and anti-rabbit secondary antibody were obtained from Santa Cruz Biotechnology (Santa Cruz, CA).

### Cell viability and colony formation assay

The cytotoxicity of matrine on A2780, SKOV3, and A2780/DDP cells were assessed by MTT assay. A2780, SKOV3, and A2780/DDP cells were seeded into 96-well plates at density of 5 × 10^3^ cells/well. After adhering to the plate, cells were exposed to matrine at various final concentrations (0, 0.25, 0.5, 1.0, 1.5, 2.0 mg/mL) for the indicated time periods (24, 48, 72 h). The absorbance at 570 nm wavelength was measured using microplate reader (Model 680, Bio-Rad, USA). Each experiment was conducted with replicates of six wells and repeated three times.

A2780 and SKOV3 cells were seeded into 6-well plates (100 cells/well) and incubated with various concentrations of matrine, respectively. After seeding for 14 days, the colonies were subsequently fixed with 4% formaldehyde, stained with 0.1% crystal violet solution (Solarbio, Beijing, China), imaged and counted. Three independent experiments were conducted.

### Cell cycle assay

A2780 and SKOV3 cells were seeded in 6-well plates and treated with the indicated concentrations of matrine for 24 h, respectively. Cells were harvested, washed, and fixed in 70% ice-cold ethanol at −20 °C overnight. On the following day, the collected cells were washed twice with PBS and treated with 100 μg/mL RNase A at 37 °C for 30 min in the dark. Propidium iodide (PI) was then added at a final concentration of 50 μg/mL for DNA staining. The distribution of cells with differing DNA content was analyzed on a FACSCalibur flow cytometer with CellQuest software (BD Biosciences, Germany).

### Apoptosis assay using Annexin V/PI staining

Cellular apoptosis rate was determined using the annexin V-fluorescein isothiocyanate (FITC) and propidium iodide (PI) apoptosis detection kit (BD Biosciences, San Diego, CA) according to the manufacturer's protocol. Briefly, A2780, SKOV3, and A2780/DDP cells were seeded in 6-well plates and exposed to matrine with different concentrations for 24 h. On the following day, cells were harvested, washed twice with cold PBS, resuspended in 500 μL binding buffer and stained with 5 μL FITC-conjugated Annexin V and 10 μL PI for 10 min at 37 °C in the darkness. Fluorescence signals from at least 10,000 cells were analyzed immediately using a FACSCalibur flow cytometry (BD Biosciences, USA). Dot plots and histograms were analyzed by FlowJo software.

### Transmission electron microscopy

A2780, SKOV3 and A2780/DDP cells were exposed to matrine for 24 h and fixed with 2.5% glutaraldehyde solution (Sigma-Aldrich, G5882) overnight, followed by the fixation with 1% OsO_4_. After dehydration in graded ethanol, 10 nm thin sections were sliced and stained with 2% uranyl acetate. Observation was performed on a JEM1230 transmission electron microscope (JEOL, Tokyo, Japan). High-resolution digital images were acquired from a randomly selected 5 different fields for samples of each condition.

### Immunostaining

A2780 and SKOV3 cells were transfected with GFP-LC3 plasmid using FuGENE^®^6 transfection reagent (Promega, E2692) for 24 h and transferred to coverslips, respectively. After transfection, cells were incubated with matrine (2.0 mg/mL) for another 24 h, and then were fixed with 4% paraformaldehyde at room temperature for 20 min. Washed 3 times with PBS, cell nuclei were stained with 4′,6-diamidino-2-phenylindole (DAPI) (Sigma-Aldrich, D9542) for 10 min. The fluorescent samples were visualized under the same parameters by a fluorescence microscope (Olympus, Japan). The images were analyzed by Image J software.

### Wound-healing and transwell invasion assay

A2780, SKOV3, and A2780/DDP cells (5 × 10^5^ cells/well) were seeded into six-well plates, and were scraped with a 200 μL sterile pipette tip when the cells achieved 90% confluence, respectively. Washed with PBS, the scratched cells were then cultured with or without matrine for another 18 h. Images were captured at the beginning (0 h) and at the end (18 h) using ×10 objectives under Inverted Microscope (Mshot, Guangzhou, China). The wound healing migration area was measured and analyzed by Image J. All the experiments were performed in triplicate.

Cellular invasion assay was performed using 24-well transwell plates (8.0 μm pore size; Corning, NY, USA). A2780 and SKOV3 cells were seeded into the upper chamber of 24-well transwell plates at a density of 5 × 10^4^ cell/well in 200 μL RPMI1640 media containing 2% FBS. The bottom chamber was filled with 500 μL completed RPMI1640 containing 10% FBS. Matrine was added to the upper chamber allowing cells to migrate for 18 h. The upper chamber was washed, fixed with 4% paraformaldehyde at room temperature for 20 min, and stained with 0.1% crystal violet (Solarbio, C8470) for 20 min for visualization. The invasion cells on the bottom of the membrane were stained, and photographs of three different fields of stained cells were captured using Inverted Microscope (Mshot, Guangzhou, China).

### Confocal immunofluorescence microscopy

A2780 and SKOV3 cells grown in laminin-coated glass coverslips were treated with or without matrine for 24 h, respectively. Cells were then fixed with 4% formaldehyde for 10 min at room temperature, permeabilized with 0.1% Triton X-100 for 5 min, and then washed 3 times with PBS. After rinsing in PBS, cells were incubated with anti-p-FAK antibodies at 4 °C overnight. On the following day, samples were then incubated with the corresponding fluorescence secondary antibody for 1 h, and actin filaments were labeled with rhodamine phalloidin for 30 min at room temperature. The coverslips were then mounted onto slides and visualized using Zeiss LSM780 confocal microscopy.

### In vitro angiogenesis assay

A2780, SKOV3 and A2780/DDP cells conditioned media (CM) were made to mimic the tumor microenvironment and the abnormalities on the blood vessel. Briefly, A2780, SKOV3, and A2780/DDP cells (60–70% confluence) were treated with matrine or PBS with indicated concentrations for 48 h. Following treatment with matrine, the treated cells were washed to remove the drug, then HUVEC-specific growth media (ECM) were added to the matrine-treated or PBS-treated A2780 and SKOV3 for 24 h to make the CM. After 24 h, the debris-free CM was collected by centrifugation and used for angiogenesis assay.

To examine the effect of matrine on in vitro angiogenesis, HUVECs were seeded into a 96-well plate (2 × 10^4^ cells/well) coated with 50 μL matrigel (Corning Incorporated, USA) and were cultured in regular media (RM) and matrine-treated or PBS-treated A2780, SKOV3 and A2780/DDP cell-derived CM. Then, cells were incubated at 37°C incubator for 4 h, and the capillary like structures were photographed by a Nikon photographic microscope. Meanwhile, VEGFA concentrations in the A2780 and SKOV3 cell culture media were determined using human VEGFA ELISA kit (Jianglaibio, Shanghai, China) according to the manufacturer’s instructions.

### Real time-PCR

The total cellular RNA was extracted from A2780 or SKOV3 cells or HUVECs using Trizol reagent (Invitrogen, Life Technologies, CA) following the manufacturer’s protocol. The concentration of the extracted RNA was determined by optical density measurement (A260/A280 ratio) using the NanoDrop system. cDNA was synthesized using HiScript^®^ II Q RT SuperMix (Vazyme Biotech Co., Ltd., Nanjing, China) following the manufacturer's instructions. Then the cDNA was amplified by real-time PCR with the ChamQ^TM^ SYBR^®^-Green qPCR Master Mix (Vazyme Biotech Co., Ltd., Nanjing, China) with the following primers (synthesized by Sangon Biotech Co., Ltd., Shanghai, China): VEGFA: forward, 5′-ATC GAG TAC ATC TTC AAG CCA T-3′; reverse, 5′-GTG AGG TTT GAT CCG CAT AAT C-3′; ANG-1: forward, 5′-GGG AGG TTG GAC TGT AAT ACA A-3′; reverse, 5′-TGT CAT ACT GTG AAT AGG CTC G-3′. The housekeeping gene glyceraldehyde phosphate dehydrogenase (GAPDH) was used for normalization (forward: 5′-TGA TGA CAT CAA GAA GGT GGT GAA G-3′; reverse: 5′-TCC TTG GAG GCC ATG TGG GCC AT-3′). Each reaction was repeated in triplicate wells. The fold-change in each sample was calculated by 2^−ΔΔCt^ method.

### Western blot

Cells were lysed in modified RIPA buffer (Thermo, Rockford, IL, USA) supplemented with protease inhibitor cocktail (Thermo, Rockford, IL, USA). Proteins obtained from cell lysates were separated on 10% SDS–PAGE by electrophoresis, and transferred to nitrocellulose membranes. The membranes were incubated with primary antibodies at 4 °C overnight, then probed with the appropriate secondary antibodies for 1 h at room temperature. The bands were visualized using an enhanced chemiluminescence detection kit (Pierce, Thermo Scientific, USA) by ChemiDoc MP Imager (BIO-RAD). Loading was normalized with GAPDH or β-actin.

### In vivo xenograft model

Female BALB/c nude mice, aged 4–6 weeks, weighed 18–22 g, were obtained from the Medical Laboratory Animal Center of Tongji Medical College and maintained with sterilized food and water. This study was carried out in accordance with the recommendations of the Guidelines for the Care and Use of Laboratory Animals, and the protocol was approved by the Institutional Animal Care and Use Committee of Tongji Medical College, Huazhong University of Science and Technology, Wuhan, China.

To investigate the suppression effect of matrine on tumor development in vivo, each mouse was injected subcutaneously with 1 × 10^6^ A2780 cells or A2780/DDP cells (100 μL in PBS) into the flank, respectively. Seven days after injection, mice were randomly divided into two groups, respectively (*n* = 3). The animals were treated with intraperitoneal injection of matrine (100 mg/kg) or normal saline three times a week. Tumor size was measured by using a caliper every 3 days and was calculated by using the following formula: Volume = 1/2 × (length × width^2^). After 28 days of cell inoculation, the mice were euthanized for assessing tumor load and tumors were collected for western blot and immunohistochemical analysis.

To examine the effect of matrine on the metastasis of ovarian cancer cell in vivo, 200 μL RPMI-1640 media containing 2 × 10^6^ A2780 cells or A2780/DDP cells were injected into the tail veins of BALB/c nude mice, and then randomly divided into two groups, respectively (*n* = 5). The following days after injection, the mice were treated with matrine (100 mg/kg, i.p.) or normal saline three times a week. After 6 weeks, mice were sacrificed and the lungs were dissected, stained with H&E and observed under a microscope (Olympus, Tokyo, Japan). The total numbers of metastatic nodules in each lung were counted in the corresponding lung sections.

### Immunohistochemistry and TUNEL assay

Tumors, hepatic tissues and pulmonary tissues were embedded in paraffin, cut into 4 μm sections, and either H&E stained or treated with corresponding antibodies for IHC evaluation. TdT-mediated dUTP nick-end labeling (TUNEL) assay was performed with one-step TUNEL apoptosis assay kit (Beyotime Institute of Biotechnology) according to the manufacturer’s instructions.

### Evaluation of ALT and AST

To assess changes in hepatic function after treatment with matrine, the whole blood of mice was collected from heart puncture using heparin-rinsed 1 mL syringes at 3 days after the last injection. The levels of serum alanine transaminase (ALT) and aspartate transaminase (AST) were evaluated to assess changes of liver function by using the Hitachi 717 Chemistry Analyzer (Hitachi, Tokyo, Japan).

### Statistical analysis

All data presented are the mean ± SD from at least three independent experiments. Comparisons between two groups for statistical significance were conducted using a Student’s unpaired *t* test. The differences between multiple groups were analyzed by one-way analysis of variance (ANOVA). *P* *<* 0.05 was considered statistically significant.
